# Modeling Hepatocellular Carcinoma Cells Dynamics by Serological and Imaging Biomarkers to Explain the Different Responses to Sorafenib and Regorafenib

**DOI:** 10.3390/cancers13092064

**Published:** 2021-04-25

**Authors:** Piero Colombatto, Coskun Ozer Demirtas, Gabriele Ricco, Luigi Civitano, Piero Boraschi, Paola Scalise, Daniela Cavallone, Filippo Oliveri, Veronica Romagnoli, Patrizia Bleve, Barbara Coco, Antonio Salvati, Lucio Urbani, Ferruccio Bonino, Maurizia Rossana Brunetto

**Affiliations:** 1 Hepatology Unit, Pisa University Hospital, 56124 Pisa, Italy; riccogabriele@gmail.com (G.R.); lcivitano@alice.it (L.C.); danielacavallone@hotmail.com (D.C.); f.oliveri@ao-pisa.toscana.it (F.O.); v.romagnoli@ao-pisa.toscana.it (V.R.); patriziableve@gmail.com (P.B.); b.coco@ao-pisa.toscana.it (B.C.); salvatiantonio@hotmail.com (A.S.); 2 Gastroenterology Department, Marmara University, 34722 Istanbul, Turkey; coskun_demirtas10@hotmail.com; 3 Radiodiagnostic Unit, Pisa University Hospital, 56124 Pisa, Italy; p.boraschi@gmail.com (P.B.); scalisepl@gmail.com (P.S.); 4 Department of Clinical and Experimental Medicine, Pisa University, 56124 Pisa, Italy; 5 General Surgery Unit, Pisa University Hospital, 56124 Pisa, Italy; l.urbani@ao-pisa.toscana.it; 6 Biostructure and Bio-Imaging Institute of National Research Council of Italy, 56124 Pisa, Italy; ferruccio.bonino@unipi.it

**Keywords:** hepatocellular carcinoma, HCC, sorafenib, regorafenib, AFP, PIVKA-II, kinetics, mathematical modeling, digital imaging

## Abstract

**Simple Summary:**

Systemic therapy in advanced hepatocellular-carcinomas (HCC) has limited benefits, but some patients show partial responses (PR) and a few even a complete response (CR). Understanding the biological mechanisms could help clinicians in decision-making. Aim of this study was to develop a physic-mathematical model to investigate tumor dynamics using α-fetoprotein (AFP) and protein induced by vitamin K absence-II (PIVKA-II) measures combined with digital imaging. The model was set-up in three prototype patients with CR/PR to sorafenib and PR to regorafenib, and then applied in seven patients with different types of response. Overall, the rate constant of cancer cells production ranged between 0.250–0.372 C × day^−1^. During therapy, neo-angiogenesis reduction was higher in four CR than in four PR or stable disease (SD) and in two non-responders (median: 83.2% vs. 29.4% vs. 2.0%). Tumor vasculature decay appeared accelerated in CR. We conclude that modeling serological and imaging biomarkers could help personalization of systemic therapy.

**Abstract:**

In advanced HCC, tyrosine-kinase inhibitors obtain partial responses (PR) in some patients and complete responses (CR) in a few. Better understanding of the mechanism of response could be achieved by the radiomic approach combining digital imaging and serological biomarkers (α-fetoprotein, AFP and protein induced by vitamin K absence-II, PIVKA-II) kinetics. A physic-mathematical model was developed to investigate cancer cells and vasculature dynamics in three prototype patients receiving sorafenib and/or regorafenib and applied in seven others for validation. Overall four patients showed CR, two PR, two stable-disease (SD) and two progressive-disease (PD). The rate constant of cancer cells production was higher in PD than in PR-SD and CR (median: 0.398 vs. 0.325 vs. 0.316 C × day^−1^). Therapy induced reduction of neo-angiogenesis was greater in CR than in PR-SD and PD (median: 83.2% vs. 29.4% and 2.0%), as the reduction of cell-proliferation (55.2% vs. 7.6% and 0.7%). An additional dose-dependent acceleration of tumor vasculature decay was also observed in CR. AFP and cancer cells followed the same kinetics, whereas PIVKA-II time/dose dependent fluctuations were influenced also by tissue ischemia. In conclusion, pending confirmation in a larger HCC cohort, modeling serological and imaging biomarkers could be a new tool for systemic therapy personalization.

## 1. Introduction

Hepatocellular carcinoma (HCC) is the sixth most common cancer and the third cause of cancer-related deaths globally [1]. Multi-modal therapeutic programs provide a significant cure rate, especially in early stage HCC [2]. However, the same treatment does not fit everybody, particularly in advanced stage HCC. The widespread lack of agreement on staging systems, prognostic scores and treatment allocation algorithms, may limit therapeutic choices. Therefore, in patients with intermediate-advanced disease difficult to frame into the Barcelona Clinic Liver Cancer (BCLC) staging, new scoring systems have been proposed [3] and better definitions of tumor biological behavior should be identified.

Sorafenib, the first tyrosine-kinase inhibitor (TKI) [4] approved for systemic treatment, is still the most used first line treatment for patients with advanced HCC [5], and regorafenib is an effective second line treatment that provided survival benefit in HCC patients progressing on sorafenib treatment [6]. Even though the median survival benefit is limited to few months, in the last decade an increasing number of patients with complete response (CR) have been described, and in some of them the response was maintained in spite of significant dose reductions due to adverse events [7,8].

A better knowledge of the mechanisms that lead to CR could increase our understanding of tumor dynamics and help clinicians in decision-making. Radiomics, combining digital imaging and serological biomarkers, represents a new study approach in which bio-mathematical modeling may boost the understanding of the processes trying to unravel the interplay of different factors [9,10,11].

We here report the development of a novel physic-mathematical model driven by digital imaging, serum α-fetoprotein (AFP) and protein induced by vitamin K absence-II (PIVKA-II) kinetics. By this approach, for the first time, cancer cells and tumor vascularization dynamics are described in a cohort of 10 patients with advanced stage HCC and different types of response to TKI.

## 2. Materials and Methods

### 2.1. Patients

Among 112 patients, who received for at least 3 months of TKI treatment for BCLC-B (33) or BCLC-C (79) HCC at the Hepatology Unit of the Azienda Ospedaliera-Universitaria Pisana from 2007, we initially identified three cases who had clinically relevant responses and sufficient imaging and serological data for developing the mathematical model: Case-1 with CR to sorafenib, Case-2 with partial response to sorafenib and Case-3 with PR to regorafenib. Their detailed clinical features and the modeling analysis are reported in the Results section. The model was then applied to fit AFP and PIVKA-II kinetics in seven other patients with different degrees of response to therapy, defined according to currently used imaging criteria [12], representing the validation cohort.

Written informed consent was obtained from all patients for the study procedures, which conformed to the ethical guidelines of the 1975 Declaration of Helsinki, as reflected in a priori approval by the institution’s human research committee.

### 2.2. Serum Biomarkers

α-Fetoprotein (AFP) was tested routinely on fresh sera by an ARCHITECT chemiluminescent-microparticle-immunoassay (Abbott, Rome, Italy). Protein induced by vitamin K absence-II (PIVKA-II) was tested retrospectively on sera stored at −20 °C since September 2015 by a quantitative fully automated chemiluminescent-enzyme-immunoassay, Lumipulse G1200 (Fujirebio Inc., Tokyo, Japan) with a dynamic range of 1.37 to 75,000 (upper normal limit 48 mAU/mL).

### 2.3. Digital Imaging Analysis

Volumetric and densitometry measurements were obtained at baseline and at each follow-up CT scan using a dedicated GE Advantage Workstation 4.6 (GE Healthcare, Inc., Waukesha, WI, USA). Volumetric assessment was performed on late arterial phase by manually tracing the lesion margins on each axial slice, with automatic calculation of the total tumor volume, (TTV) (cm^3^) by the software. The same workstation was used to obtain densitometry measurements by tracing a region of interest (ROI) within the lesion in unenhanced and each post-contrastographic scans. A tumor vascularization index (TVI) was defined by the difference between the average density measured in Hounsfield units (HUs) during the arterial phase in the tumor mass and in non-tumor liver.

### 2.4. Mathematical Model

The full description of the model, with the assumptions and the procedures used to compute the parameters, is provided in the Supplementary Materials.

## 3. Results

### 3.1. Model Set-Up in Prototype Patiens

#### 3.1.1. Case-1 (CR to Sorafenib)

**Figure 1:**
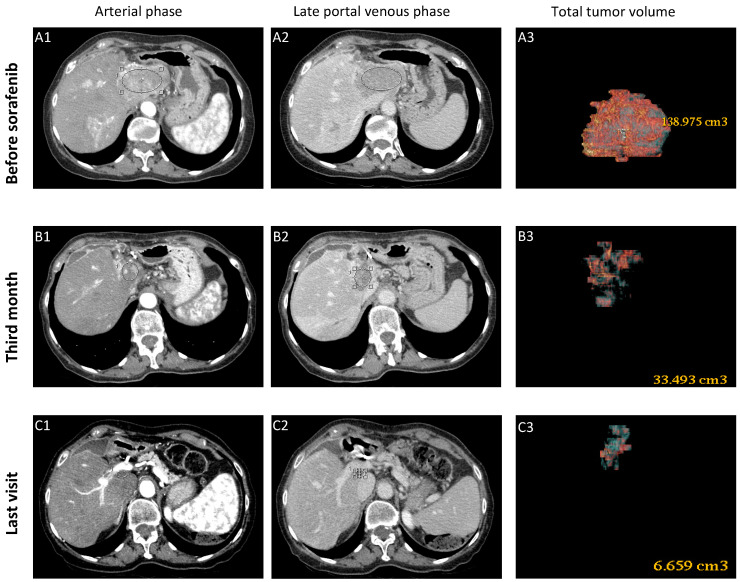
Baseline CT scan (**A1**,**A2**) showing the large HCC mass (maximum diameter 75 mm) in the left liver lobe with contrast enhancement in the arterial phase and wash-out in the late portal phase with vascular invasion. Follow-up CT scans demonstrated the markedly reduced sized necrotic lesion (maximum diameter 32 mm) at the third month of therapy (**B1**,**B2**) that remains barely noticeable (maximum diameter 18 mm) at the last visit (**C1**,**C2**), with no contrast enhancement in both phases. Total tumor volume (TTV) declined from 138.9 cm^3^ (**A3**) to 33.4 cm^3^ (**B3**) in 3 months, and to 6.6 cm^3^ at the last visit (**C3**) after 48 months from the beginning of therapy.

In May 2001, a 65-year-old Caucasian woman with chronic hepatitis B (CHB) and Child-Pugh Score (CPS) A cirrhosis, was referred to our unit with a 15 mm nodule in liver segment VII detected at the ultrasound surveillance. The abdomen computed tomography (CT) confirmed the lesion as a well-differentiated HCC (BCLC stage 0); Eastern Cooperative Oncology Group performance status (ECOG PS) was 0. AFP values, that were high (243 ng/mL) because of liver regeneration following a recent ALT flare (461 U/L) of her untreated HBeAg negative CHB, dropped to normal shortly thereafter, when nucleoside analogue (lamivudine) treatment was started. For HCC treatment, the patient underwent four sessions of percutaneous ethanol injection (PEI) between January and February 2002 with CR at the control CT, but in February 2010, after eight years of radiologic remission, magnetic resonance imaging (MRI) detected a novel 13 mm HCC in segment IV, and she underwent six sessions of PEI with CR. In June 2013, local recurrence was detected in segment IV with normal AFP, and two additional sessions of PEI were performed. However, 6 months later, CT demonstrated the increase in the size of the segment IV lesion to a diameter of 25 × 15 mm with invasion to the proximal section of the left portal vein, AFP was 12.4 ng/mL. HCC was classified as advanced stage (BCLC C, CPS A, ECOG PS 0) due to the portal vein invasion. The patient underwent trans-arterial radioembolization (TARE) in April 2014, achieving a radiologic remission with disappearance of the neoplastic portal vein thrombosis for approximately two years. In a follow-up control in January 2016, despite the negative MRI performed four months earlier, a sharp increase in both AFP and PIVKA-II serum levels occurred (AFP: 2377 ng/mL; PIVKA-II: 30362 mAU/mL). CT scan showed the appearance of 75 × 45 mm sized HCC in the left lobe with invasion to the main trunk of portal vein and hepatic veins. For the first time, the patient was symptomatic, complaining of fatigue and weight loss. She was considered to have an advanced stage HCC (BCLC C, CPS A, ECOG PS 2) and she was started on sorafenib 400 mg/day. A striking radiologic response was observed at 3 months (Figure 1), accompanied by partial regression of vascular invasion.

AFP and PIVKA-II levels declined to normal range within 8 and 12 months, respectively. The clinical condition of the patient improved and she gained 8 kg in 6 months, even with drug-related side effects (grade 3 diarrhea with abdominal distension, fatigue, hitching and alopecia). Her maximum tolerated dose of sorafenib (600 mg/day) was maintained only for 1 month, till July 2016, when it was diminished to the initial dose (400 mg/day). Then, side effects of fluctuating intensity (grade 2–3) allowed her to assume variable doses (on average 300 mg/day) until July 2017, when the dose was decreased at 200 mg/day. Since October 2017 the dose of 200 mg was taken every other day with minimal side effects. Follow-up CTs demonstrated the progressive reduction of the tumor burden with consistent shrinkage of the left lobe and complete regression of the vascular invasion. Based on modified RECIST2 criteria [12] a CR was achieved after 15.5 months of therapy, when no arterial phase contrast enhancement was documented in a residual lesion of 32 mm in diameter. At the last CT performed on February 2021, the residual area of the lesion was about 15 mm in diameter, yet without arterial contrast enhancement.

**Figure 2:**
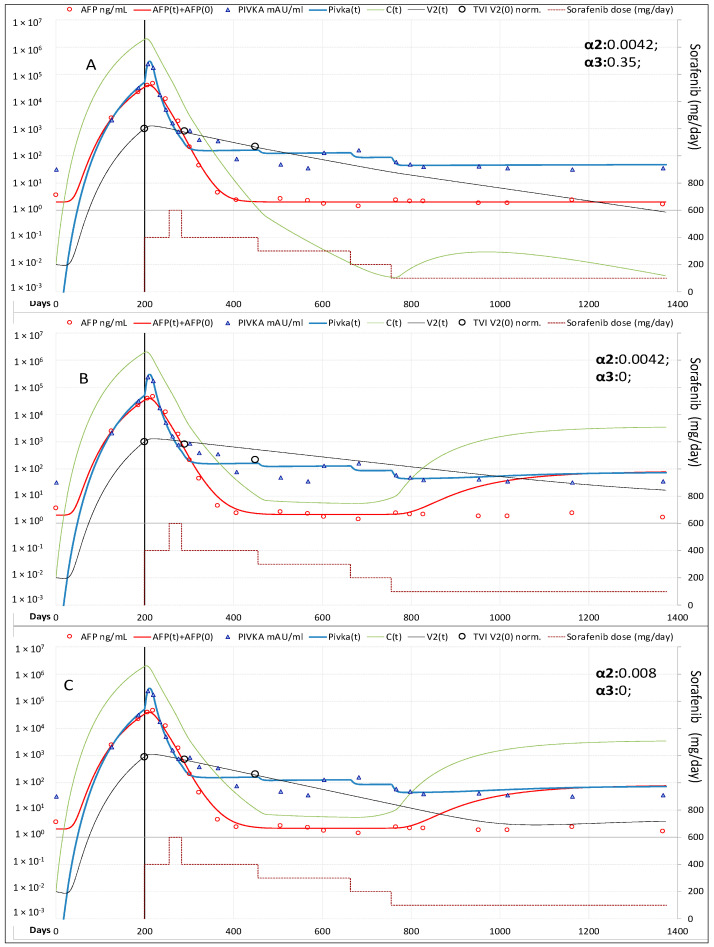
Model fitting of measured variables in Case-1. (**A**) Best fitting of Tumor Vascular Index (TVI), AFP and PIVKA-II serum levels. (**B**) In absence of the additional dose dependent decay constant of tumor vasculature, the model predicts recurrence of HCC when sorafenib dose is reduced to 200 mg every other day. (**C**) Halving the mean lifetime of tumor vascularization the model still predicts tumor recurrence. *Legend*: AFP = measured AFP; AFP(t) = model computed AFP; AFP(0) = AFP normal value; PIVKA-II = measured PIVKA-II; Pivka(t) = computed PIVKA-II; C(t) = model computed cancer cells; V2(t) = model computed vascularization index; TVI V2(0) norm = CT measured tumor vascular index normalized to V2 at treatment baseline.

Best fitting of experimental data before therapy was reached by setting the cancer cells C(t) daily rate production equal to 0.360 × C(t)^0.931^, with the exponent <1 indicating that new cancer cells produced by a single cancer cell decreased as the tumor mass increased; C(t) decay at a daily rate equal to 0.11 × C(t), corresponding to cancer cells mean life-time of 9.1 days. AFP was produced at a daily rate of 0.0022 × C(t) and decayed at a daily rate equal 0.10 × AFP(t), corresponding to AFP mean lifetime of 10.0 days. PIVKA-II was produced at a daily rate of 0.00025 × C(t)^1.22^, with the exponent >1 indicating that PIVKA-II produced by a single cancer cell increases as the tumor mass increases, and decayed at a daily rate equal to 0.2 × PIVKA-II(t) corresponding to a mean lifetime of 5.0 days. The number of cancer cells present at the beginning of treatment C(0) = 1.78 × 10^6^ cells/mL was estimated from the TTV measured at the baseline CT (139 cm^3^), under the assumption that HCC cells have on average the same volume of normal hepatocytes and occupy 50% of TTV. The time required for reaching that volume resulted to be 174 days. Steady-state plasma levels of sorafenib F(t), comparable to those reported in pharmacokinetics studies [13] of approximately 0.01 mg/mL at the daily oral dose of 400 mg bid, were reached setting the F(t) daily yield rate equal to 0.000013 × D(t), where D(t) is the daily intake of sorafenib (mg/die). F(t) decayed at a daily rate equal to 0.50 × F(t), corresponding to a mean life-time of 2.0 days. During the first weeks of therapy AFP continued to increase but at a slower rate. For this reason, a time delay between sorafenib bioavailability and actual drug effectiveness [F(t)_del_] on the hepatocytes was assumed. In the same period, PIVKA-II levels showed a spike (Sp) preceding its rapid decline, and, later on, fluctuations not correlated to C(t). Such complex kinetics could depend from the anti-vascular effects of this drug on cancer cells and/or from their toxicity [Tox(t)] on non-neoplastic hepatocytes [14,15,16,17]. Fitting of PIVKA-II levels was reached indeed by computing values of F(t)_del_, Sp(t) and Tox(t) as described in Supplementary Materials. The effectiveness of sorafenib in reducing neo-angiogenesis was defined by the model parameter ε_υ_ = 1/[1 + ϑ1 × F(t)_del_], the value of ϑ1 = 220 was obtained by best-fitting of C(t) and PIVKa-II decline, which yielded a 77.4% reduction once reached the steady state of drug activity (about 3 weeks after treatment start). The effectiveness of sorafenib in reducing cancer cells replication was defined by the model parameter ε_r_ = 1/[1 + ψ1 × F(t)_del_], the value of ψ1 = 10 was obtained by AFP fitting and yielded a 13.5% decline once reached the steady state of drug activity. By fitting CT scans measured TVI in the first 9 months of therapy, we calculated the decay constant of tumor vasculature (α2 = 0.0042, which yielded a vasculature mean lifetime of 238 days) However, to acknowledge the fact that HCC did not recur despite lowering the doses of sorafenib in the following years (Figure 2), the vasculature lifetime had to be reduced setting the parameter α3 = 0.35, thus attributing to the drug the possibility to accelerate vasculature degradation, in addition to that yet described of reducing neo-angiogenesis. According to this hypothesis, the vasculature lifetime was only 23 days with a sorafenib dose of 400 mg qd.

#### 3.1.2. Case-2 (PR to Sorafenib)

**Figure 3:**
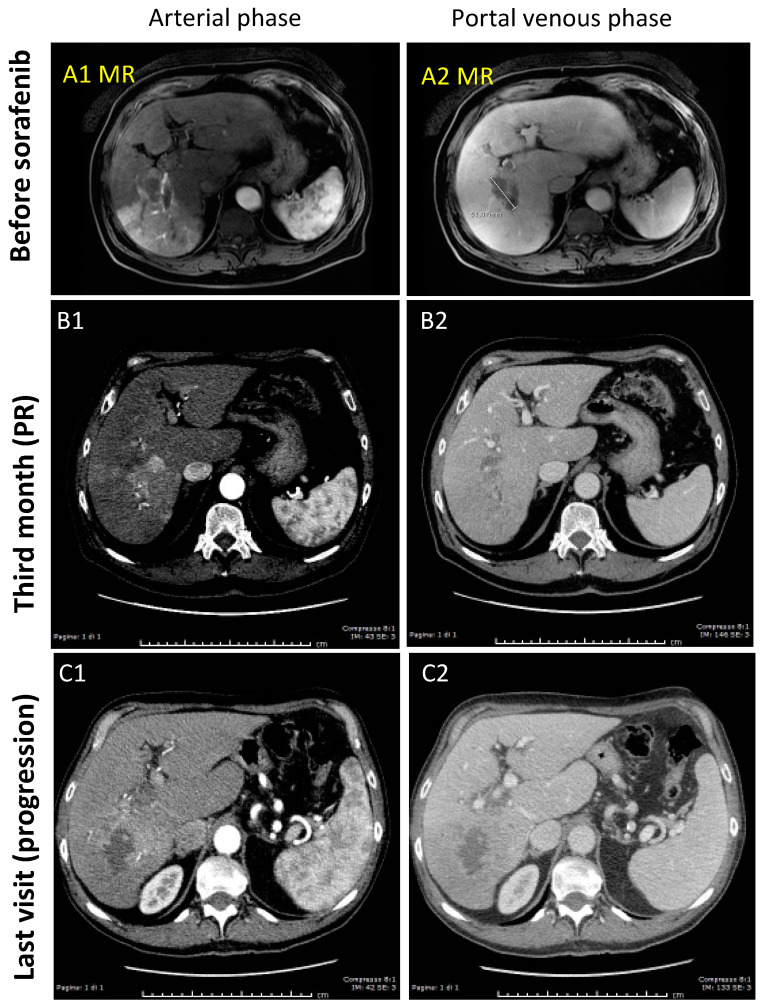
Baseline MRI (**A1**,**A2**) showing a widespread hypo-vascular HCC in liver segment IV, with complete thrombosis of the portal branch for the VII–VI liver segments. A partial response after 3 months of sorafenib therapy was documented by CT scans (**B1**,**B2**), which has been progressively lost, as shown at the last CT scan performed after 20 months of therapy (**C1**,**C2**).

In 2012, a 72-year-old Caucasian man with Child-A cirrhosis, due to prior chronic active hepatitis C and type 2 diabetes, was referred for un unexpected increase of AFP (563 ng/mL) five years after successful treatment of HCV with peg-interferon and ribavirin. In the CT scan performed 6 months earlier no neoplastic lesions were detected, but the MRI performed after AFP elevation (Figure 3) showed the appearance of a widespread HCC in liver segment IV, with aspects of infiltrative growth and almost complete thrombosis of the portal branch for the VII-VI liver segments and partial thrombosis of the right portal branch. He was considered to have an advanced stage HCC (BCLC C, CPS A, ECOG PS 1) and he was started on sorafenib 800 mg/day. He had a partial response to therapy, but eventually the disease progressed, as shown at the last CT scan performed after 20 months of therapy. The patient died 28 months from the beginning of therapy. The progression was, at least in part, attributable to sorafenib dose reductions imposed by side effects, as also suggested by AFP and PIVKA-II kinetics.

**Figure 4:**
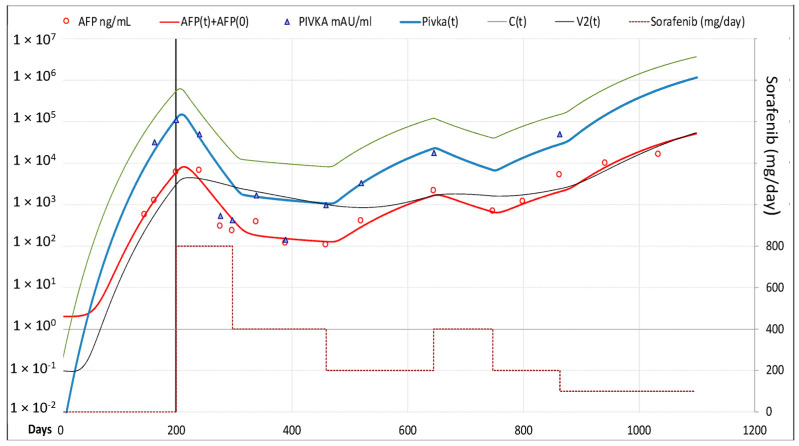
Best fitting of AFP and PIVKA-II serum levels in Case-2. AFP and PIVKA-II levels showed fluctuations correlated to C(t) and to the dose of sorafenib. *Legend*: AFP = measured AFP; AFP(t) = model computed AFP; AFP(0) = AFP normal value; PIVKA-II = measured PIVKA-II; Pivka(t) = computed PIVKA-II; C(t) = model computed cancer cells; V2(t) = model computed vascularization index.

Model computed cancer cells daily rate production was 0.250 × C(t)^0.959^, and C(0) = 3.17 × 10^6^ cells/mL. AFP was produced at the daily rate of 0.0015 × C(t); PIVKA-II at the daily rate of 0.0010 × C(t)^1.15^, and its mean lifetime was 3.3 days. Best fitting of C(t), AFP and PIVKA-II decline was obtained setting ϑ1 = 50 and ψ1 = 5, which yielded a 50.9% and 9.4% reduction in neo-angiogenesis and cancer cells replication, respectively. Both AFP and PIVKA-II levels showed fluctuations correlated to C(t), and best fitting was reached with Sp(t) and Tox(t) equal to 0 (Figure 4).

#### 3.1.3. Case-3 (PR to Regorafenib)

**Figure 5:**
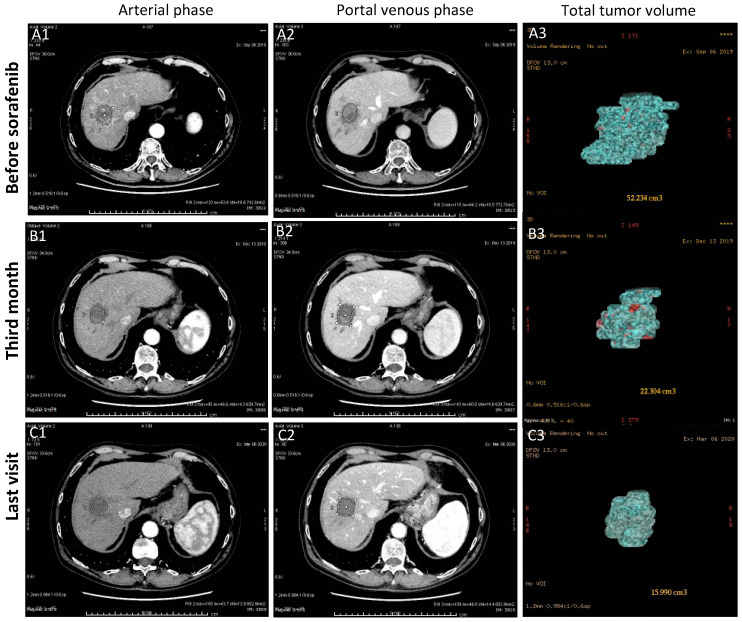
Baseline CT scan (**A1**,**A2**) showing the hypo-vascular nodule of HCC of 65 × 45 mm involving segment V-VII-VIII with satellites and thrombosis of the right portal branch. A slow partial response to regorafenib therapy was documented after 3 months (**B1**,**B2**), which still continues after 12 months of therapy (**C1**,**C2**), as confirmed by the progressive reduction of the tumor volume (**A3**–**C3**).

In 2019, upon accidental finding of esophageal varices (stage F1) by upper GI endoscopy performed because of dyspepsia, a 70-year-old Caucasian man was diagnosed with liver cirrhosis due to genetic hemochromatosis. Further investigations, including liver imaging and biochemistry, revealed the presence of Child-A cirrhosis with very high AFP levels (29,953 ng/mL). The CT scan (Figure 5) pointed out a single hypo-vascular nodule of HCC of 65 × 45 mm involving segment V-VII-VIII with satellites and thrombosis of the right portal branch. He was considered to have an advanced stage HCC (BCLC C, CPS A, ECOG PS 1) and he was started on sorafenib 400 mg/day, but one month later he was switched to regorafenib 160 mg/day because CT showed thrombosis progression and AFP levels up to 46,255 ng/mL. A partial response to regorafenib was documented by CT scan after 3 months and continued thereafter. Treatment is still ongoing and it is associated to a slow reduction of the tumor volume and of AFP levels.

**Figure 6:**
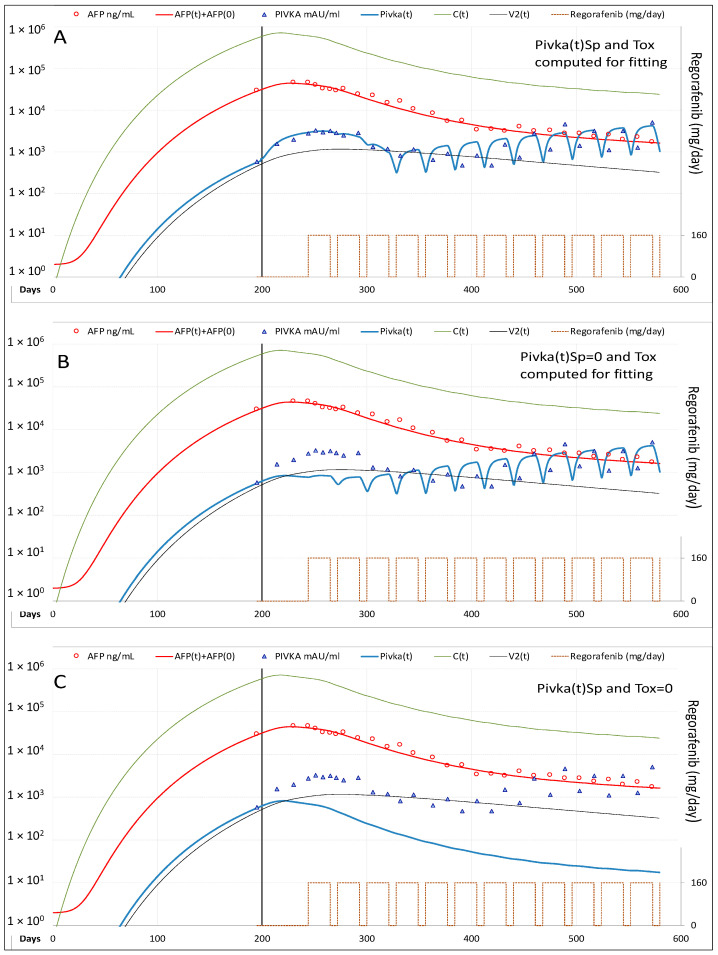
Model fitting of measured variables in Case-3 (**A**) Best fitting of AFP and PIVKA-II serum levels. AFP decline well correlated to C(t) decline, whereas PIVKA-II levels showed more complex kinetics, which appear influenced by the schedule of regorafenib treatment. The increase of PIVKA-II production observed in the first 3 months was fitted including in the model the potential effect of ischemia induced by the drug on cancer cells [Pivka(t) Sp]. The later PIVKA-II behavior, characterized by rapid fluctuations, was strongly influenced by the treatment schedule; this behavior could be explained considering that regorafenib may also exerted anti-vascular/toxic effects on non-tumor liver cells, that led to the production of a certain amount of PIVKA-II [Pivka(t) Tox]. (**B**) Best fitting of AFP and PIVKA-II obtained without including Pivka(t) Sp. (**C**) Best fitting of AFP and PIVKA-II obtained without including both Pivka(t) Sp and Pivka(t) Tox. *Legend*: AFP = measured AFP; AFP(t) = model computed AFP; AFP(0) = AFP normal value; PIVKA-II = measured PIVKA-II; Pivka(t) = computed PIVKA-II; C(t) = model computed cancer cells; V2(t) = model computed vascularization index.

By modeling cancer cells daily rate production was 0.32 × C(t)^0.932^, and C(0) = 5.79 × 10^5^ cells/mL. AFP was produced at the daily rate of 0.0065 × C(t); PIVKA-II at the daily rate of 0.00007 × C(t)^1.14^, and its mean life-time was 2.5 days. Best-fitting of C(t), AFP and PIVKA-II decline was obtained setting ϑ1 = 30 and ψ1 = 5, which yielded a 27.1% and 5.8% reduction in neo-angiogenesis and cancer cells replication, respectively. Best fitting of PIVKA-II levels was achieved computing the values of Sp(t) and Tox(t) (Figure 6) according to the methods reported in Supplementary Materials.

Briefly, the reduction of PIVKA-II plasma levels during effective TKI therapy can be preceded by a transient increase, attributed to the ischemia of cancer cells [15]. The amount of PIVKA-II produced in the spike was computed assuming that it is proportional to PIVKA-II value at the beginning of therapy P(0) and to the density (R) of the cancer cells in the tumor mass. The increase may have a temporal delay and kinetics different from those of cancer cells. In addition, these drugs can also exert anti-vascular toxic effects on non-tumor liver cells [18,19]; the hypothetical amount of PIVKA-II produced in this way, expressed by the term Tox(t), was computed according to the equation Tox(t) = π4 × F(t) × t^π5^, where the coefficient π4 is the parameter that accounts for the dose dependent effect (no toxicity in Case-2 = 0; in Case-3 = 15), and the exponent π5 accounts for the time dependent effect (in Case-3 = 1.52). In this patient, the fluctuations observed after 3 months of therapy are due to the PIVKA-II Tox(t) component, and reflect the dose of active drug F(t) which fluctuates because of the treatment schedule adopted with regorafenib, given every day for 3 weeks followed by one week off (Figure 6).

### 3.2. Model Validation Cohort

#### 3.2.1. Clinical Characteristics of the Patients

The model was applied to fit the kinetics of AFP and PIVKA-II in seven patients who received sorafenib for advanced stage HCC (one BCLC-B and six BCLC-C), mainly after failure of prior local treatments (Table 1).

According to imaging-based mRECIST criteria [12], three patients showed CR of the target lesion: Case-4 underwent orthotropic liver transplant (OLT) after 11.5 months and remained disease free thereafter, whereas Case-5 and Case-6 developed new HCC lesions at different sites after 25.7 and 18.4 months of therapy, showing overall disease progression (PD). Case-7 and Case-8 showed stable disease (SD) of the original tumor, but overall PD due to the appearance of new lesions after 9.3 and 18.9 months of therapy. In the remaining two patients (Case-9 and Case-10) the target lesions did not show any appreciable response to sorafenib, which was withdrawn after 5.1 and 5.7 months.

Demographic, clinical and oncological characteristics of all patients analyzed by the model are reported in Table 1.

#### 3.2.2. Fitting of AFP and PIVKA-II Serum Levels

**Figure 7:**
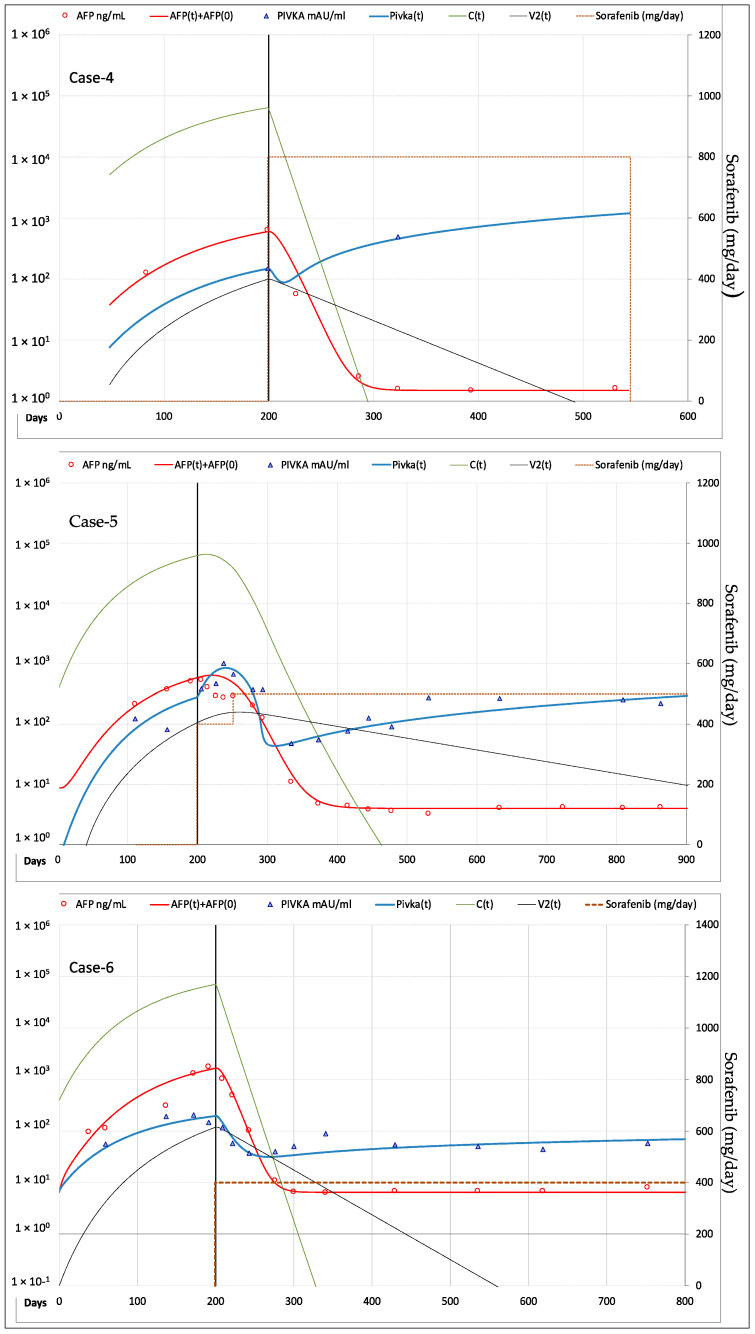
Best fitting of AFP and PIVKA-II serum levels in the 3 patients with CR of the target lesion. In Case-4 the modeling analysis is stopped when sorafenib treatment was discontinued and patient underwent liver transplantation. In Case-5 and Case-6 modeling analysis is stopped when they developed new HCC lesions at different sites after 25.7 and 18.4 months of therapy. *Legend*: AFP = measured AFP; AFP(t) = model computed AFP; AFP(0) = AFP normal value; PIVKA-II = measured PIVKA-II; Pivka(t) = computed PIVKA-II; C(t) = model computed cancer cells; V2(t) = model computed vascularization index.

**Figure 8:**
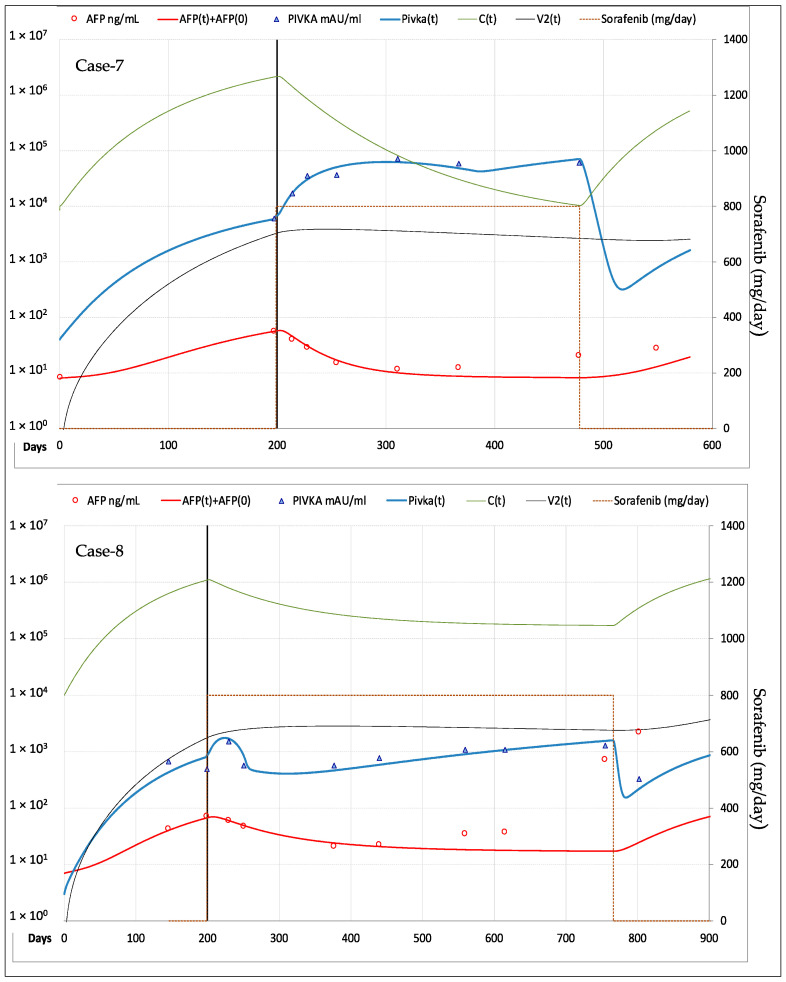
Best fitting of AFP and PIVKA-II serum levels in the 2 patients with SD of the target lesion, but overall progression due to the appearance of new lesions after 9.3 and 18.9 months of therapy. *Legend*: AFP = measured AFP; AFP(t) = model computed AFP; AFP(0) = AFP normal value; PIVKA-II = measured PIVKA-II; Pivka(t) = computed PIVKA-II; C(t) = model computed cancer cells; V2(t) = model computed vascularization index.

**Figure 9:**
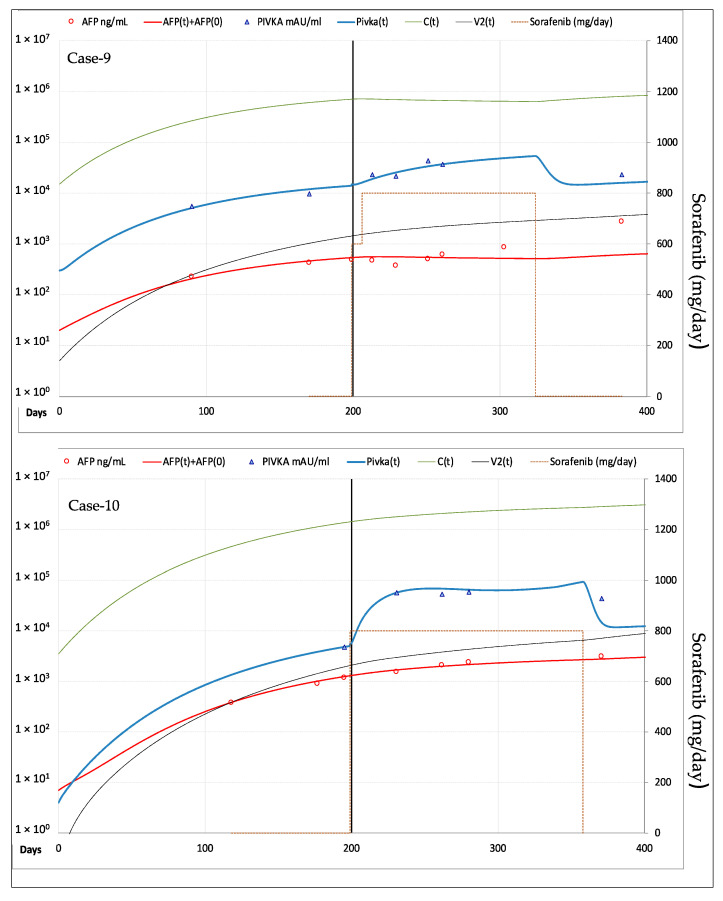
Best fitting of AFP and PIVKA-II serum levels in the 2 patients with PD of the target lesion. Case-9 showed a minimal initial response in term of AFP reduction. *Legend*: AFP = measured AFP; AFP(t) = model computed AFP; AFP(0) = AFP normal value; PIVKA-II = measured PIVKA-II; Pivka(t) = computed PIVKA-II; C(t) = model computed cancer cells; V2(t) = model computed vascularization index.

Fitting of AFP and PIVKA-II in the patients forming the validation cohort is shown in Figure 7 for the three cases who had CR of the target lesion, in Figure 8 for the two cases with SD and in Figure 9 for the remaining two cases with PD. The values of the most relevant model parameters used for fitting AFP and PIVKA-II levels are reported in Table 2.

The median rate of cancer cell production (ξ1) was numerically higher in PD than in PR-SD and CR (0.398 vs. 0.325 vs. 0.316 C × day^−1^), whereas the decay constant of cancer cells was similar in all patients (0.11–0.12 day^−1^). The exponent ξ2, describing the C(t) dependent efficiency of cell replication, was computed with limited experimental data before therapy, therefore not reliable for further analysis. The median rate constant of AFP production by cancer cells (ω1) was numerically lower in PD than in PR-SD and CR (0.0002 vs. 0.0008 vs. 0.0016 C × day^−1^), whereas the median decay constant of AFP (0.10–0.12 day^−1^) was not different among the three groups and similar in all patients but one (Case-7, who showed the lowest AFP production rate and low AFP serum levels). The median rate of PIVKA-II production by cancer cells (π1) was numerically higher in PD than in PR-SD and CR (0.0041 vs. 0.0015 vs. 0.0002 C × day^−1^). The C(t) dependent production efficiency (π2) and the decay constant of PIVKA-II (π3) were similar in the three groups.

The coefficient (μ1), describing increase of the drug (D) in plasma after 1 mg/day intake, showed median values 3-fold higher in CR, as compared to PR-SD and PD patients (0.000022 vs. 0.000008 and 0.000007 D × day^−1^). The decay constant of plasma active drug (μ2) was similar in all patients (0.4–0.5 day^−1^). Drug anti-vascular effectiveness (ϑ1) was greater in CR (median: 185, range: 100–500) than in PR-SD (median: 30, range: 13–50) and PD (median: 2, range: 1–3), which translates into a median reduction of neo-angiogenesis of 83.2%, 29.4% and 2.0%, respectively. Similarly, the anti-replicative effectiveness (ψ1) was greater in CR (median: 30, range: 10–70) than in PR-SD (median: 5, range: 3–10) and PD (median: 0.5, range: 0–1), which translates into a median reduction of cancer cells proliferation of 55.2%, 7.6% and 0.7%, respectively.

The decay constant of tumor vasculature (α2) was similar in all patients (0.0020–0.0042 day^−1^). Best fitting of the experimental data required the additional drug dependent vasculature decay constant (α3) in all CR patients (median: 0.275, range: 0.200–0.400) and in 2 PR-SD patients (0.002–0.27). The values of the remaining parameters are reported in Table S1 of the Supplementary Materials where the full description of the physic-mathematical model is available.

## 4. Discussion

In this work we describe a novel physic-mathematical model that allowed us to investigate the biological mechanisms of response to TKI in advanced HCC with elevation of AFP and PIVKA-II levels, two of the most studied and validated serological biomarkers already used in clinical practice [20].

CR to sorafenib is rare, and remains unclear which mechanisms are involved and whether treatment can be withdrawn [7,8]. Sorafenib is an inhibitor of several signal pathways including RAF/ERK/MERK, c-MET, vascular endothelial growth factor receptor (VEGFR) and platelet-derived growth factor receptor (PDGFR), with a great potential in biologically heterogeneous tumors [4], but its efficacy in HCC is unpredictable, heterogeneous and not easy to evaluate. Currently, the uptake of contrast agent in the arterial phase of dynamic CT or MRI, is the reference method to assess the persistence of viable tumor in the lesion [2,12], while serum biomarkers, such as AFP and PIVKA-II, can integrate the evaluation [14,15,16,17]. To better investigate the correlations between imaging response and HCC biomarkers kinetics, we developed a model of tumor dynamics with a set of ordinary differential equations that was used to fit measured AFP and PIVKA-II serum levels, according to basic biological assumptions and known mechanisms of action of sorafenib and regorafenib. Our model was set up by a data driven approach in three cases: Case-1 who had CR to sorafenib, Case-2 with dose-dependent PR to sorafenib, and Case-3 who has PR to the ongoing treatment with regorafenib. Once established, the model was applied in a small validation cohort of 7 patients with different types of response, as reported in Table 1.

A striking feature of Case-1 was the rapid growth of the HCC when it recurred after trans-arterial radioembolization. In fact, the MRI performed 124 days before diagnosis could not detect any lesion. By model fitting of pre-treatment AFP and PIVKA-II levels, we computed the mean lifetime of cancer cells (9.1 days) and their production rate during the initial stage (0.36/day), finding an average cancer cells doubling time of 2.7 days. According to previous models [11], the rates of cancer cells proliferation and vasculature daily production are not constant but tend to decrease at the increasing of the tumor mass. Under these conditions, the model calculated that cancer growth occurred in 174 days to reach the TTV of 149 cm^3^ measured at the beginning of therapy. Tumor volume doubling time computable using these parameters was about 3.7 days during the initial phase and about 27 days in the period immediately preceding therapy, which is consistent with the findings obtained in the imaging studies [21]. In the two cases with PR, the rate constant of cancer cells production was slightly lower: 0.25/day in Case-2, yet consistent with the absence of detectable lesions in a CT scan performed 8 months earlier, and 0.32/day in Case-3, for whom no prior imaging was available. The average cancer growth estimated in these cases appears to be fast, although within the wide range reported in experimental studies [21,22]. This finding is probably explained by the selection bias introduced enrolling only patients with advanced disease to be treated with systemic therapy, therefore, such grow rates may not apply to the HCCs diagnosed in the earlier stages of the BCLC classification.

Considering the whole cohort of 10 patients analyzed (Table 2), the daily cancer cells production rate constant ranged between 0.250 and 0.440, standing in the higher part of the range for the 2 patients with PD who did not show any significant response to sorafenib (0.440 and 0.355). However, since the number of cases is small and this variable appears to be rather heterogeneous in HCCs, this finding can just raise the hypothesis of an inverse relationship between the tumor growth rates and response to TKI, which deserves further studies in larger cohorts. The decay constant of cancer cells was instead similar in all patients, suggesting that the average lifetime of HCC cells is a much less heterogeneous biological variable. Other parameters estimated by the model resulted highly heterogeneous among patients, in particular the average daily rate of AFP and PIVKA-II production by cancer cells, which encompassed a 3 Log range in this cohort. These findings are not surprising taking into account that up to 40–50% of HCCs do not have elevated levels of AFP [20]. By contrast the decay constant of AFP (0.1–0.3 day^−1^) and PIVKA-II (0.2–0.5 day^−1^) showed a limited variability among patients.

Interestingly, early after the beginning of sorafenib treatment, a further increase of PIVKA-II not correlated with AFP and cancer cells kinetics was observed in most of the cases. Elevations of PIVKA-II levels during the first month of treatment were reported previously and associated with prolonged time to progression, suggesting that PIVKA-II early kinetics could help to predict treatment response. However, the decrease of AFP only was correlated with good response [14,15,16,17]. Model analysis pointed out some differences between the two biomarkers: the mean life-time of PIVKA-II (2.0–5.0 days) was shorter than that of AFP (9.1–10.0 days in all cases but one), the latter being closer to that of cancer cells (9.1 days). These findings suggest that PIVKA-II levels are less directly correlated than AFP levels to the proliferation of cancer cells, depending also on other conditions, such as tumor vascularization.

The spike of PIVKA-II levels, well documented during the first month of therapy in Case-1, Case-5 and Case-8, could be attributed to the block of angiogenesis induced by sorafenib that up-regulates PIVKA-II levels through ischemia of HCC cells [15,16,17]. In fact, hypoxia altering the actin molecules making up their cytoskeleton impairs the endocytosis of vitamin K, and the subsequent vitamin K deficiency leads to the release into the circulation of vitamin K deficient prothrombin without complete carboxylation [23]. On the other hand, PIVKA-II appears to be also involved in neo-angiogenesis [24] and able to act as a mitogen stimulating the growth of HCC cells [25], thus contributing to the increased risk of HCC in cirrhotic patients [26,27]. Altogether, these data support the interpretation that PIVKA-II production by cancer cells is increased by ischemia and tend to stimulate angiogenesis.

In Case-3, however, the increase of PIVKA-II was slower and prolonged for 3 months, then followed by rapid fluctuations strongly correlated with the schedule of regorafenib dosing, that is administered consecutively for 3 weeks followed by one week off treatment. Since hypoxia is a condition frequently found within the cores of tumors [28] but also in chronic liver diseases of different etiologies, in particular when cirrhosis is present [29], we hypothesize that regorafenib could have exerted its anti-vascular toxic effects on non-tumor liver cells also [19,20]. In fact, best fitting of PIVKA-II levels in Case-3 (Figure 6) was obtained assuming that a mechanism similar to that causing the increase of PIVKA-II production by cancer cells can also affect non-neoplastic hepatocytes with different dose and time dependent relationships.

Accordingly, the complex PIVKA-II kinetics were successfully fitted by the model describing the early spike of PIVKA-II as a consequence of the drug anti-vascular effects on cancer cells and the later one, observed in almost all cases, due to a similar mechanism acting on non-neoplastic hepatocytes. These findings support the necessity of a modeling analysis to interpret the discrepancies between AFP and PIVKA-II kinetics at the single patient level.

Another important issue that could be addressed by modeling is whether sorafenib treatment is still effective at low doses and/or can be withdrawn in the few patients with consolidated CR. To this aim, it should be noted that to acknowledge the fact that HCC did not recur despite significant lowering of the dose in Case-1 required an additional drug induced decay constant for the existing vasculature (α3). At the steady state, with a dose of 400 mg qd, the model ascribes to sorafenib antineoplastic activity the abatement of neo-vascularization down by 77.4%, and of cancer cells proliferation down by 13.5%. However, such relevant effects were not sufficient to explain the persistence of the response after dose reductions. The lack of recurrence could be explained setting α3 = 0.350, meaning that the drug further reduced cancer cells production by reducing the average lifetime of the existing tumor vasculature from 238 days to 23 days. This mechanism was negligible in the first months of therapy but became relevant when the dose was reduced at 200 mg q2d (Figure 2).

Interestingly, to achieve the best fitting of the experimental data the additional drug dependent vasculature decay constant was required in all CR patients (median: 0.275, range: 0.200–0.400) and in 2/4 PR-SD patients (0.002–0.27). However, the major differences between patients with or without response to the TKI, were related to the parameters describing drug effectiveness, in terms of anti-angiogenesis and of anti-replicative activity. Indeed, a median reduction of neo-angiogenesis of 83.2%, 29.4% and 2.0% was observed in CR, PR-SD and PD, respectively. Similarly, the median reduction of cancer cells proliferation was 55.2%, 7.6% and 0.7%, respectively. These findings are consistent with the mechanism of action of these drugs and modeling could be used to anticipate or integrate imaging criteria for the evaluation of the response. Such approach would have been particularly useful in patients with lesser drug efficacy (i.e., Case-8, Case-9 and Case-10), where the divergence of AFP measured levels from the model predicted values let hypothesize the selection and expansion of more aggressive HCC clones that could benefit from a second line treatment.

Another interesting difference pointed out by modeling regards the coefficient (μ1) describing plasma increase of the drug (D) after 1 mg/day intake, which showed median values 3-fold higher in CR, as compared to PR-SD and PD patients. Overall, these findings suggest that TKI pharmacokinetics could have a role in explaining, at least in part, the different responses observed in our patients. In addition, in responders to sorafenib, the lower doses might target more the maintenance of existing vasculature than neo-angiogenesis. Accordingly, in cell-based assays, sorafenib was able to block autophosphorylation of VEGFR-2, VEGFR-3, PDGFR, Flt-3, and c-KIT at significantly lower concentrations (20 to 100 nmol/L) than those required for inhibition of the RAF/MEK/ERK pathway (90 to 4000 nmol/L) [4]. However, such interpretation requires confirmation by further experimental observations and an even more complex modeling approach [30,31], that it is not feasible with the data available from patients treated in real-life clinical practice. We must also recognize that, because of the technical limits in studying quantitatively tumor vascularization by routine CT scan procedures, the relationship between tumor vascularization and cancer cells proposed in the model was evaluated in Case-1 only, by approximated fittings of the TVI measured by CT with the model computed vascular index (V2). In addition, it is not possible to establish whether the acceleration of the natural decay of the existing vasculature, suggested by the modeling analysis in CR, was due solely to sorafenib or to other concomitant conditions. In Case 1, for instance, tumor shrinkage was accompanied by complete atrophy of the left liver lobe, and we cannot rule out that the left branch portal vein thrombosis (Figure 1) and the previous TARE may have played a role in tumor de-vascularization obtained by systemic treatment [32,33]. Overall, portal vein thrombosis was present in half of the patients analyzed, and we cannot exclude the possibility that changes of liver hemodynamics could have interfered on model parameters.

Besides the intrinsic points of weakness discussed above, the small sample size is the principal limitation of the study. Indeed, our work needs further validation in larger cohort of patients to prove that the kinetics of PIVKA-II and AFP, analyzed by the model here proposed, could better quantify the different antineoplastic activities in all patients receiving TKIs. Another finding that deserves further investigation is that in patients in whom sorafenib showed a greater effectiveness in blocking neo-angiogenesis, the drug appeared effective at lower doses also, accelerating the tumor vasculature decay. This additional activity was introduced to explain the persistence of the response in Case-1 after dose reductions, and it is in agreement with the findings of Rimola et al. [8] who reported in 11 patients with CR the recurrence of the tumor in 5/7 of those who discontinued, versus 0/4 of those who continued on treatment.

## 5. Conclusions

In conclusion, we here provided the proof-of-concept of a novel approach to study the variability of the response to TKIs, which stems from the heterogeneity of tumor biology and drug individual susceptibility, by developing a physic-mathematical model able to interpret in a small cohort of patients the kinetics of PIVKA-II and AFP, combined with digital imaging. Pending confirmation in larger series of cases, this approach could allow for a more accurate evaluation of therapy efficacy in clinical practice and may represent a new tool for future studies aimed to better characterize the response with systemic therapies.

## Figures and Tables

**Table 1:** Demographic, clinical and oncological characteristics of the patients enrolled.

Clinical Features	Model Set-Up			Model Validation						
	Case 1	Case 2	Case 3	Case 4	Case 5	Case 6	Case 7	Case 8	Case 9	Case 10
Gender	F	M	M	M	F	M	M	M	M	M
Age at treatment start	79	72	70	65	64	56	76	61	67	69
Liver disease etiology	HBV	HCV	GH	HBV	HCV	HBV	HCV	HBV	HCV	HCV
HCC Staging (BCLC)	C	C	C	B	C	C	B	C	C	C
Prior HCC treatments	PEI, TARE	-	-	TACE	TACE	RFTA	TACE	-	TACE, PEI	-
HCC volume (cm^3^)	139	32	52.2	6.4	24.5	6.1	200	89	29.1	116
Vascular invasion	Yes	Yes	Yes	No	Yes	No	No	Yes	Yes	Yes
Lymph-node mts	No	No	No	No	No	Yes	No	No	No	No
AFP at BL (ng/mL)	21,531	5905	29,953	635	513	1741	55	71	417	1167
PIVKA at BL (AI/mL)	30,362	108,460	592	151	388	147	6000	589	9799	4712
Treatment	SOR	SOR	RGR	SOR	SOR	SOR	SOR	SOR	SOR	SOR
Duration (months)	60	27	12	11.5	25.7	18.4	9.3	18.9	5.1	5.7
Target Response *	CR	PR	PR	CR	CR	CR	SD	SD	PD	PD
Overall Response *	CR	PD	PR	CR **	PD	PD	PD	PD	PD	PD

Legend: F: Female; M: Male; HBV: Hepatitis B Virus; HCV: Hepatitis C Virus; GH: Genetical Hemochromatosis; HCC: Hepatocellular Carcinoma; BCLC: Barcelona Clinic Liver Cancer; PEI: Percutaneous Ethanol Injection; TARE: Trans-Arterial Radio-Embolization; TACE: Trans-Arterial Chemo-Embolization; RFTA: Radio- Frequency Thermal- Ablation; mts: metastasis; AFP: α-fetoprotein; BL: Baseline; PIVKA: Protein Induced by Vitamin K Absence; SOR: Sorafenib; RGR: Regorafenib; CR: Complete Response; PR: Partial Response; SD: Stable Disease; PD: Progression Disease. * Response according to mRECIST; ** No recurrence after liver transplantation

**Table 2:** Model parameters computed by best fitting of AFP and PIVKA-II levels in all patients.

Response *	Model Set-Up			Model Validation						
	CR	PR	PR	CR	CR	CR	SD	SD	PD	PD
Model Parameter	Case 1	Case 2	Case 3	Case 4	Case 5	Case 6	Case 7	Case 8	Case 9	Case 10
ξ1	0.360	0.250	0.320	0.317	0.315	0.315	0.360	0.330	0.440	0.355
ξ2	0.931	0.959	0.932	0.917	0.910	0.911	0.924	0.926	0.90	0.923
ξ4	0.11	0.11	0.11	0.12	0.11	0.11	0.11	0.11	0.11	0.11
ω1	2.20 × 10^−3^	1.50 × 10^−3^	6.50 × 10^−3^	1.00 × 10^−3^	2.00 × 10^−4^	3.00 × 10^−3^	7.00 × 10^−6^	6.00 × 10^−6^	3.00 × 10^−4^	1.00 × 10^−4^
ω2	0.10	0.10	0.10	0.10	0.10	0.12	0.30	0.10	0.10	0.10
π1	2.50 × 10^−4^	1.00 × 10^−2^	7.00 × 10^−5^	2.00 × 10^−4^	2.00 × 10^−4^	9.80 × 10^−2^	3.00 × 10^−3^	5.00 × 10^−5^	8.00 × 10^−3^	2.00 × 10^−4^
π2	1.22	1.15	1.14	1.14	1.20	0.62	0.88	1.13	1.00	1.14
π3	0.2	0.3	0.4	0.4	0.4	0.5	0.20	0.4	0.4	0.4
μ1	1.30 × 10^−5^	1.30 × 10^−5^	7.00 × 10^−6^	3.00 × 10^−5^	5.00 × 10^−6^	9.00 × 10^−5^	8.00× 10^−6^	7.00 × 10^−6^	7.00 × 10^−6^	7.00 × 10^−6^
μ2	0.5	0.5	0.4	0.4	0.4	0.4	0.4	0.4	0.4	0.4
ϑ1	220	50	30	500	150	100	30	13	3	1
α2	4.20 × 10^−3^	4.20 × 10^−3^	2.00 × 10^−3^	4.00 × 10^−3^	2.00 × 10^−3^	2.00 × 10^−3^	2.00 × 10^−3^	2.00 × 10^−3^	2.00 × 10^−3^	2.00 × 10^−3^
α3	0.350	0.270	0.002	0.200	0.400	0.200	0	0	0	0
ψ1	10	5	5	10	70	50	10	3	1	0

Legend. Response of the target lesion according to mRECIST *; CR: Complete Response; PR: Partial Response; SD: Stable Disease; PD: Progression Disease. ξ1: Rate constant of cancer cells production; ξ2: C(t) exponent in cancer cells daily production; ξ4: Decay constant of cancer cells; ω1: Rate constant of AFP production by single cancer cell; ω2: Decay constant of plasma AFP; π1: Rate constant of PIVKA-II production by single cancer cell; π2: C(t) exponent in PIVKA-II daily production; π3: Decay constant of plasma PIVKA-II; μ1: Coefficient of F(t) daily increase by 1 mg/day of D(t); μ2: Plasma F(t) decay constant; ϑ1: Drug anti-vascular effectiveness; α2: Decay constant of tumor vasculature; α3: Additional F(t) dependent vasculature decay constant; ψ1: Drug anti-replicative effectiveness.

## Data Availability

Authors agree to make data and materials supporting the results presented in this paper available upon reasonable request.

## Supplementary Materials

The following are available online at https://www.mdpi.com/article/10.3390/cancers13092064/s1, Full description of the physic-mathematical model. Table S1: Description and values of the model parameters used for fitting AFP and PIVKA-II levels in the 10 patients analysed.
