# Supplementary material for: Modeling Hepatocellular Carcinoma Cells Dynamics by Serological and Imaging Biomarkers to Explain the Different Responses to Sorafenib and Regorafenib
Source: Cancers (Basel). 2021 Apr 25;13(9):2064. doi: 10.3390/cancers13092064 (PMC8123288; doi:10.3390/cancers13092064)
Supplement: Supplementary file 1 [file cancers-13-02064-s001.zip › cancers-1189953-supplementary.pdf]

Supplementary Materials: Full description of the physic-mathematical model

Modeling hepatocellular carcinoma cells dynamics by serological and imaging biomarkers to explain complete response to sorafenib

Piero Colombatto[1], Coskun Ozer Demirtas[2], Gabriele Ricco[1,6], Luigi Civitano[1], Piero Boraschi[3], Paola Scalise[3], Daniela Cavallone[1,6], Filippo Oliveri[1], Veronica Romagnoli[1], Patrizia Bleve[1], Barbara Coco[1], Antonio Salvati[1], Lucio Urbani[4], Ferruccio Bonino[5] and Maurizia Rossana Brunetto[1,5,6].

Affiliations:

1) Hepatology Unit, Pisa University Hospital, Pisa, Italy;

2) Gastroenterology Department, Marmara University, Istanbul, Turkey;

3) Radiodiagnostic Unit, Pisa University Hospital, Pisa, Italy;

4) General Surgery Unit, Pisa University Hospital, Pisa, Italy;

5) Biostructure and Bio-imaging Institute of National Research Council of Italy, Italy;

6) Department of Clinical and Experimental Medicine, Pisa University, Pisa, Italy.

Corresponding author:

Prof. Maurizia Rossana Brunetto,

Hepatology Unit of the Pisa University Hospital and Department of Clinical and Experimental Medicine, University of Pisa, Pisa, Italy.

Via Paradisa 2 – Cisanello, 56124, Pisa, Italy

Tel: +39-050-9956857

Fax: +39-050-995457

Mail: maurizia.brunetto@unipi.it

Table of contents

Model description

a) *Number of cancer cells present at the beginning of sorafenib treatment*

b) *Sorafenib pharmacokinetics*

c) *Dynamics of tumor vascularization*

d) *Dynamics of cancer cells*

e) *Dynamics of AFP*

f) *Dynamics of PIVKA-II*

Computation of model parameters

Supplementary Table 1

Model description

Here following we report the assumptions, the parameters and the mathematical expressions used to model the dynamics of AFP and PIVKA-II serum levels before and during sorafenib treatment.

a) *The number of cancer cells present at the beginning of sorafenib treatment* was estimated for Case-1 by CT scan measured total tumor volume (TTV) under the assumption that HCC cells have the same volume of normal hepatocytes ($Vc = 19.7 \cdot 10^{-9} cm^3$) and occupy 50% of TTV (70 cm^3). Thus the number of cancer cells at therapy baseline was $3.55 \cdot 10^9 cells$. For consistency with AFP and PIVKA-II, the number of cancer cells (*C*) is referred to 1 mL of plasma. Assuming plasma volume of 2000 mL, the normalized number of cancer cells at therapy baseline is $C(0) = 1{,}78 \cdot 10^6\ cells/mL$.

b) *Sorafenib pharmacokinetics* was described by the following equation:

$$\frac{dF(t)_{fast}}{dt} = \mu 1 \cdot D(t) - \mu 2 \cdot F(t) \qquad [1]$$

$F(t)_{fast}$ is plasma drug concentration (mg/mL), *D(t)* the daily dose of drug taken (mg/day); $\mu 1$ (mL^{-1}) is the daily increase of *F(t)* induced by 1 mg/day of the drug, and $\mu 2$ (day^{-1}) the decay constant of the drug.

To account for the time delay *(del)* between sorafenib bioavailability and actual effectiveness of drug on the hepatocytes, the value $F(t)_{del}$ was calculated by the following equation:

$$F(t)_{del} = n \cdot t^m \cdot F(t)_{fast} \qquad [2]$$

The values of the parameters n and m are obtained by best fitting of AFP behavior during the first weeks of therapy, under the condition $0 \le n \cdot t^m \le 1$, where $t = t - t_0$ (t_0 = time when the current dose started). In correspondence of any changes of dose, the effective drug concentration $F(t)_{del}$ is given by:

$$F(t)_{del} = F(t_-)_{del} + \lfloor F(t_+)_{fast} - F(t_-)_{fast} \rfloor \cdot n \cdot t^m \qquad [3]$$

c) *Dynamics of tumor vascularization* were described according to the evidence that sorafenib is primarily an inhibitor of tumor angiogenesis. Thus, drug dependent inhibition of tumor vascularization (ε_v) is defined by the following expression, where the parameter $\vartheta 1$ represents the effectiveness of the drug:

$$\varepsilon_v = \frac{1}{1 + \vartheta 1 \cdot F(t)_{del}} \qquad [4]$$

Since the daily increase of angiogenesis $[dV1(t)/dt]$ depends on daily increase of cancer cells *dC(t)/dt*, and *dC(t)/dt* depends on *C(t)* [Eq. 8], we defined a tumor Vascularization index *V1(t)* that changes during therapy as a function of *C(t)* according to the following equation:

$$\frac{dV1(t)}{dt} = \alpha 1 \cdot \varepsilon_v \cdot C(t) - \alpha 2 \cdot V1(t) \qquad [5]$$

The term $\alpha 1$ (day^{-1}) is the rate constant of tumor angiogenesis induced by a single cancer cell, whereas $\alpha 2$ (day^{-1}) represents tumor vasculature decay constant.

Since sorafenib could affect not only angiogenesis but also the vitality of the vasculature already present in the tumor, a second vascularization index *V2(t)* was introduced to take into account this potential effect of the drug. The equation describing *V2(t)* is:

$$\frac{dV2(t)}{dt} = \alpha 1 \cdot \varepsilon_v \cdot C(t) - [\alpha 2 + \alpha 3 \cdot F(t)_{del}] \cdot V2(t) \qquad [6]$$

The term $\alpha 3 \cdot F(t)_{del}$ represents the additional vasculature decay constant induced by the drug, dependent on its effective plasma concentration $F(t)_{del}$.

d) *Dynamics of cancer cells* were described assuming that sorafenib inhibits the proliferation of cancer cells by affecting neo-angiogenesis, accelerating tumor vasculature decay and reducing cell replication. The coefficient accounting for direct inhibition of cancer cells replication (ε_r) is defined by the following expression, where the parameter $\psi 1$ represents the effectiveness of the drug:

$$\varepsilon_r = \frac{1}{1 + \psi 1 \cdot F(t)_{del}} \qquad [7]$$

Thus, the overall description of cancer cells dynamics is written as follows:

$$\frac{dC(t)}{dt} = \xi 1 \cdot \varepsilon_r \cdot \varepsilon_v \cdot C(t)^{\xi 2} \cdot \left(\frac{V2(t)}{V1(t)}\right)^{\xi 3} - \xi 4 \cdot C(t) \qquad [8]$$

The term $\xi 1$ (day^{-1}) is the rate constant of daily cancer cells production in absence of therapy, the exponent $\xi 2$ accounts for changes in replication efficiency dependent on the number of cancer cells (with $\xi 2 < 1$ tumor growth slows down at the increasing of the tumor mass). The term $\left(\frac{V2(t)}{V1(t)}\right)^{\xi 3}$ describes the inhibitory effects of sorafenib on the new and the existing vasculature; $\xi 3$ takes into account the influence of the ratio *V2(t)/V1(t)* on *C(t)* replication. The term $\xi 4$ is the rate constant of the cancer cells decay (day^{-1}).

e) *Dynamics of AFP* are correlated to those of cancer cells $[dC(t)/dt]$, which are a function of *C(t)* [Eq 8], according to the following equation:

$$\frac{dAFP(t)}{dt} = \omega 1 \cdot C(t) - \omega 2 \cdot AFP(t) \qquad [9]$$

The production rate constant of AFP by one cancer cell is described by the term $\omega 1,$ and $\omega 2$ represents plasma AFP decay constant (day^{-1}).

f) *Dynamics of PIVKA-II.* The whole dynamics of PIVKA-II plasma levels (*P*) were fitted by the following equation:

$$\frac{dP(t)}{dt} = \pi 1 \cdot C(t)^{\pi 2} + Sp(t) + Tox(t) - \pi 3 \cdot P(t) \qquad [10]$$

The parameter $\pi 1$ represents the rate constant of PIVKA-II production, the exponent $\pi 2$ accounts for a nonlinear relationship between the increase of cancer cells and the increase of PIVKA-II production, and $\pi 3$ indicates the PIVKA-II decay constant (day^{-1}).

Since the reduction of PIVKA-II plasma levels during effective sorafenib therapy can be preceded by a transient increase due to its anti-vascular effects, the amount of PIVKA-II produced in the spike (*Sp*) was computed assuming that this increase is proportional to PIVKA-II value at the beginning of therapy *P(0)* and to the density (*R*) of the cancer cells in the tumor mass.

$$Sp(t) = \Sigma 1 \cdot P(O) \cdot R \cdot [Z(t) - Z(O)] \qquad [11]$$

Where $\Sigma 1$ is a multiplication factor that defines the amplitude of the spike. The density of cancer cells (R) represents the ratio of cancer cells over the whole cells present in the tumor mass at baseline [$C_{(0)} / H_{(0)}$]. The parameter $Z(t)$ is calculated by the following expression:

$$\mathrm{Z(t)} = \frac{exp\frac{t - t_0 - \Sigma 2}{\Sigma 3}}{1 + exp\frac{t - t_0 - \Sigma 2}{\Sigma 3 \cdot \Sigma 4}} \qquad [12]$$

Where $\Sigma 2$ is a temporal delay parameter (days), $\Sigma 3$ is a time constant (days), and $\Sigma 4$ is a distortion factor of the sigmoid generated by the expression above. For computed values of $Sp(t) < 0$, the value of $Sp(t) = 0$ in equation 10.

Since the drug can also exert anti-vascular toxic effects on non-tumor liver cells, the amount of PIVKA-II produced in this way is expressed by the term $Tox(t)$ and computed as following:

$$Tox(t) = \pi 4 \cdot F(t) \cdot t^{\pi 5}$$

The coefficient $\pi 4$ accounts for a dose dependent toxicity, whereas the exponent $\pi 5$ for a time dependent effect.

Computation of model parameters

In Case-1, fitting of AFP(t) and PIVKA-II(t) experimental data before therapy with AFP(0) = 2 ng/mL as normal value (not produced by cancer cells), and computing the number of cancer cells at the beginning of sorafenib treatment $C(0)$ by TTV, allowed us to determine the parameters: $\omega 1 = 2.2 \cdot 10^{-3}$; $\omega 2 = 0.1$, [Eq 9]; $\pi 1 = 2.5 \cdot 10^{-4}$; $\pi 2 = 1.22$; $\pi 3 = 0.20$, [Eq 10]; $\xi 1 = 0.36$; $\xi 2 = 0.931$; $\xi 4 = 0.11$, [Eq 8].

Under these conditions the time required to reach the TTV measured at the beginning of therapy was 174 days. Steady-state $F_{fast}(t)$ plasma levels comparable to those reported in pharmacokinetics studies, approximately 10 ug/mL at the daily oral dose of 200 mg bid, were reached setting the coefficient $\mu 1 = 1.3 \cdot 10^{-5}$. Sorafenib decay constant $\mu 2$ was 0.5 day^{-1}.

During sorafenib therapy, the value of $\vartheta 1$ [Eq. 4] and $\psi 1$ [Eq. 7] was computed by finding the decline of C(t) which provides the best-fitting of AFP decline ($\vartheta 1 = 2.2 \cdot 10^2$ and $\psi 1 = 10$). The value of the Vascularization index at the beginning of therapy was normalized as *V1(0)* = 1000 by setting the parameter $\alpha 1 = 1.77 \cdot 10^{-5}$. The value of the parameter $\alpha 2 = 0.0042$ [Eq. 5] (vasculature mean life-time equals to 238 days) was obtained by fitting *V2(t)* to the CT scan based estimates of tumor vascularization, here defined Tumor Vascular Index (TVI). Such index was computed by the difference between the average arterial phase density (expressed in Hounsfield Units) in the tumor mass and in non-tumor liver measured at baseline and after 88 and 248 days of therapy. TVI baseline value was normalized to 1000 arbitrary units, thus on therapy TVI values declined to 815 and 231, respectively, while *V2(t)* calculated at the same time points by best fitting of AFP and PIVKA-II were 758 and 213, respectively. The additional dose dependent decay constant of tumor vasculature $\alpha 3 = 0.35$ was set to acknowledge the fact that HCC did not recur despite lowering sorafenib doses.

The values of model parameters obtained as described above for fitting AFP and PIVKA-II levels in in all Cases are reported in the Supplementary Table 1.

Supplementary Table 1. Description and values of the model parameters used for fitting AFP and PIVKA-II levels in the 10 patients analysed.

		Model set up cases			Validation cohort						
Therapy response of the target lesion (mRECIST)		CR	PR	PR	CR	CR	CR	SD	PD	PD	PD
**Model parameters**	**Symbol**	**Case 1**	**Case 2**	**Case 3**	**Case 4**	**Case 5**	**Case 6**	**Case 7**	**Case 8**	**Case 9**	**Case 10**
Number of Cancer cells at the beginning of Treatment	**CTo**	1.78E+06	5,32E+05	5,79E+05	6,41E+04	6,22E+04	7,01E+04	2.10E+06	1.09E+06	7.06E+05	1,42E+06
Coefficient of *F(t)* daily increase by 1 mg/day of *D(t)*	**μ1**	1.30E-05	1.30E-05	7.00E-06	3,00E-05	5,00E-06	9,00E-05	8.00E-06	7.00E-06	7.00E-06	7.00E-06
Plasma *F(t)* decay constant	**μ2**	0,5	0.5	0.4	0,4	0,4	0.4	0.4	0.4	0.4	0.4
F(t) dependent delay of drug effectiveness	***n***	0.008	0.06	0.008	0.4	0.001	0.08	0.8	0.08	0.08	0.008
Time dependent delay of drug effectiveness	***m***	1.6	1.6	1.5	0.6	1.5	1.5	1.5	1.5	1.5	1.5
Drug anti-vascular effectiveness	**ϑ1**	220	50	30	500	150	100	30	13	3	1
C(t) dependent tumor angiogenesis rate constant	**α1**	1.77E-05	2.50E-04	2.50E-05	1.77E-05	1.77E-05	1.77E-05	1.77E-05	1.77E-05	1.77E-05	1.77E-05
Decay constant of tumor vasculature	**α2**	4,20E-03	4,20E-03	2,00E-03	4,00E-03	2,00E-03	2,00E-03	2,00E-03	2,00E-03	2,00E-03	2,00E-03
Additional *F(t)* dependent vasculature decay constant	**α3**	0.350	0.270	0.002	0.200	0.400	0.200	0	0	0	0
Drug anti-replicative effectiveness	**ψ1**	10	5	5	10	70	50	10	3	1	0
Rate constant of cancer cells production	**ξ1**	0.360	0.250	0.320	0.317	0.315	0.315	0.360	0.330	0.440	0.355
C(t) exponent in cancer cells daily production	**ξ2**	0.931	0.959	0.932	0.917	0.910	0.911	0.924	0.926	0.90	0.923
V2(t) / V1(t) exponent on *C(t)* replication	**ξ3**	0.4	0	0	0.4	1	0	1	0	0	0
Decay constant of cancer cells	**ξ4**	0.11	0.11	0.11	0.12	0.11	0.11	0.11	0.11	0.11	0.11
Rate constant of AFP production by *C(t)*	**ω1**	2,20E-03	1,50E-03	6,50E-03	1,00E-03	2,00E-04	3,00E-03	7,00E-06	6,00E-06	3,00E-04	1,00E-04
Decay constant of plasma AFP	**ω2**	0.10	0.10	0.10	0.10	0.10	0,12	0.30	0.10	0.10	0.10
Rate constant of PIVKA-II production by *C(t)*	**π1**	2,50E-04	1,00E-02	7,00E-05	2,00E-04	2,00E-04	9,80E-02	3,00E-03	5,00E-05	8,00E-03	2,00E-04
C(t) exponent in PIVKA-II daily production	**π2**	1.22	1.15	1.14	1.14	1.20	0,62	0,88	1.13	1.00	1.14
Decay constant of plasma PIVKA-II	**π3**	0.2	0.3	0.4	0.4	0.4	0.5	0,20	0.4	0.4	0.4
Multiplication factor defining PIVKA-II spike amplitude	**Σ1**	3	3	12	1	5	0	15	13	100	40
Cancer cells density within the tumor mass	***R***	1	0	1	1	1	0	1	1	0.5	0.3
Temporal delay factor	**Σ2**	5	5	40	0	40	0	0	0	0	15
Time constant	**Σ3**	3.5	3.5	70	70	40	2	70	30	100	20
Distortion factor	**Σ4**	0.75	0.75	0.6	0.8	0.55	0.7	0,8	0.75	0.7	0.85
Dose dependent toxicity	**π4**	1800	0	15	35	14	30	300	6	20	35
Time dependent toxicity	**π5**	0.1	1,00E-09	1.52	0.93	1,1	0.4	1.4	1.4	1.4	2.2
